# The Straw That Broke the Camel’s Back: Amoxicillin-Clavulanate Unraveling Autoimmune Hepatitis in a Previously Asymptomatic Female

**DOI:** 10.7759/cureus.91240

**Published:** 2025-08-29

**Authors:** John A Gallagher, Yara Dababneh, Andrew Gregory, Beena U Ahsan, Humberto C Gonzalez

**Affiliations:** 1 Internal Medicine, Wayne State University School of Medicine, Detroit, USA; 2 Gastroenterology and Hepatology, Henry Ford Health System, Detroit, USA; 3 Pathology, Henry Ford Health System, Detroit, USA

**Keywords:** acute liver failure, amoxicillin-clavulanate, autoimmune hepatitis, drug-induced liver injury, histopathology, immunosuppressive therapy

## Abstract

Drug-induced liver injury (DILI) is a challenging diagnosis. Treatment generally involves cessation of the offending agent. In rare cases, DILI may present with autoimmune features that may necessitate immunosuppressive treatment, as in drug-induced autoimmune hepatitis (DIAIH). Distinguishing DIAIH from classic idiopathic autoimmune hepatitis (cAIH) may be difficult, as cAIH can also present after DILI and typically requires long-term immunosuppression for treatment. We describe a case of a 61-year-old female who presented with jaundice and abnormal liver function tests several months after taking amoxicillin-clavulanate. A comprehensive workup with long-term monitoring later revealed the cause of her condition, an unusual diagnosis in a previously healthy individual.

## Introduction

Drug-induced liver injury (DILI) is a rare, yet potentially fatal cause of acute liver failure (ALF) [1]. Many drugs cause idiosyncratic (spontaneous) DILI, with antibiotics being the most frequently reported [2]. Among them, amoxicillin-clavulanate (AC) is the most common [3,4]. deLemos et al. [5] described 117 patients with DILI attributed to AC. In these patients, latency from drug exposure to presentation ranged from weeks to months, liver injury patterns were either hepatocellular or cholestatic, histopathology often showed immuno-allergic features, and individuals generally recovered fully [5]. DILI may also rarely result in drug-induced autoimmune hepatitis (DIAIH) or uncover classic autoimmune hepatitis (cAIH) in susceptible individuals [6,7]. In the United States, the prevalence of autoimmune hepatitis (AIH) is reported to be 31.2 per 100,000 individuals, with females, Caucasians, and those with concurrent autoimmune conditions such as celiac disease, rheumatoid arthritis, and inflammatory bowel disease being more commonly affected [8]. Up to 20% of cases of AIH have been estimated to be due to DIAIH specifically [6,9].

Though distinguishing between these entities may be challenging, doing so is important. cAIH is more likely to relapse and is associated with a higher risk of advanced fibrosis and cirrhosis [6,7]. Consequently, cAIH also more frequently requires long-term immunosuppressive therapy than DIAIH [6,7]. There are other notable differences between these conditions. For instance, DIAH tends to present within days of drug exposure, while cAIH typically presents months following the use of an implicated drug [6]. DIAIH is also commonly self-limited once the identified offending agent is withdrawn, with only a short-term of low-dose steroids typically being necessary [10]. Other clinical features, such as an elevated antinuclear antibody (ANA) titer and biopsy findings like inflammatory plasma cell infiltrate, are also more suggestive of the diagnosis of cAIH than DIAIH [10,11].

Many drugs have been implicated in DILI with cAIH, most commonly nitrofurantoin, minocycline, and infliximab [6,7]. However, AC-induced DILI with AIH requiring long-term immunosuppression has rarely been reported in the literature [12,13]. Zhan et al. [12] described a suspected case of AC-induced DILI, which required long-term immunosuppressive treatment and laboratory monitoring for suspected AIH, but was not confirmed by liver biopsy. In a similar case of probable AIH due to AC presented by Bariş Kuzu et al. [13], while a liver biopsy was reported, our case is unique for a longer latency between AC exposure and DILI, two relapses requiring long-term immunosuppression over a year after the initial diagnosis, and the use of well-accepted clinical diagnostic tools for definitive diagnosis of AIH. In summary, we present a unique case

## Case presentation

A 61-year-old female with a history of type 2 diabetes mellitus, hypertension, dyslipidemia, and Hashimoto’s thyroiditis presented to the emergency department for symptoms of scleral and skin yellowing, dark urine, pale stools, and abdominal pain ongoing for four weeks. She denied a personal history of liver disease, alcohol or recreational drug use, and recent sick contacts. Upon presentation, her vital signs and physical exam were normal apart from jaundice. Her labs on presentation were notable for significantly elevated liver enzymes (aspartate aminotransferase (AST) 506 IU/L and alanine aminotransferase (ALT) 583 IU/L), bilirubin (total 10 mg/dL and direct 7.4 mg/dL), and alkaline phosphatase (267 IU/L), with a normal INR of 1.0, as shown in Table 1; these values represented an R factor > 5, consistent with liver injury of a hepatocellular pattern [14]. Her medications for her chronic conditions, namely, metformin and simvastatin, were subsequently held; she was admitted for further workup, and hepatology was consulted for further evaluation.

**Table 1:** The patient’s pertinent lab values upon presentation Values in bold are elevated.

Lab Test	Value	Reference Range
Alanine Aminotransferase (IU/L)	583	0-44
Aspartate Aminotransferase (IU/L)	506	0-40
Alkaline Phosphatase (IU/L)	267	39-117
Bilirubin, Total (mg/dL)	10	0-1.2
Bilirubin, Direct (mg/dL)	7.4	0-0.4
International Normalized Ratio	1.0	0.8-1.2

The patient also had an extensive series of serologic studies. Testing for viral hepatitis, herpes simplex virus, Epstein-Barr Virus (EBV), and cytomegalovirus was unremarkable, and autoimmune studies, including anti-mitochondrial (AMA) and smooth muscle antibodies (SMA), were normal apart from a highly elevated ANA titer (1:640) with centromere pattern (Table 2).

**Table 2:** The patient’s pertinent viral and autoimmune studies Values in bold are elevated.

Lab Test	Titer/Value	Reference Range
Antinuclear Antibody (ANA)	1:640	<1:160
ANA Pattern	Centromere	Negative
Anti-mitochondrial Antibody (AMA)	<1:20	<1:20
Smooth Muscle Antibody (SMA)	<1:20	<1:20
Hepatitis A Antibody, IgM	Nonreactive	Nonreactive
Hepatitis B Core Antibody, IgM	Nonreactive	Nonreactive
Hepatitis B Surface Antigen	Nonreactive	Nonreactive
Hepatitis C Virus Antibody	Nonreactive	Nonreactive
Herpes Simplex Virus 1	Negative	Negative
Herpes Simplex Virus 2	Negative	Negative
Epstein-Barr Virus, IgM	<0.2 Antibody Index (AI)	≤ 0.8 AI
Cytomegalovirus, IgM	<0.2 AI	≤ 0.8 AI

**Figure 1:**
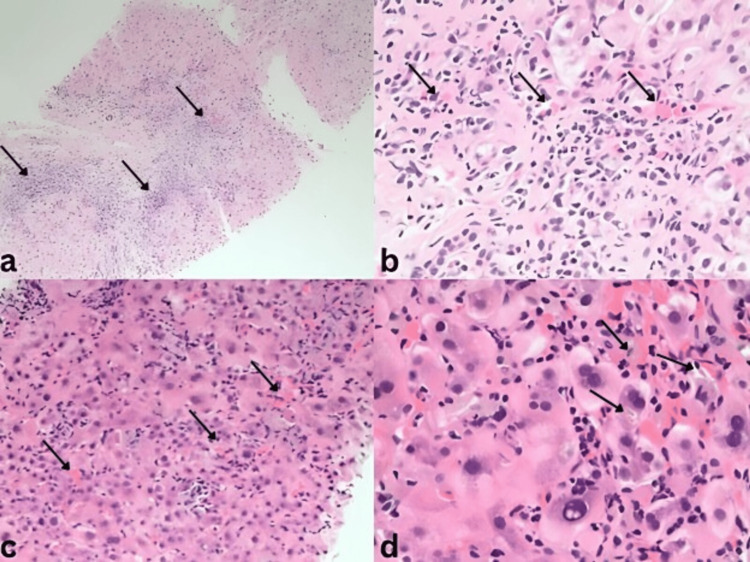
Representative histopathologic findings of liver injury due to amoxicillin-clavulanate a) The portal tracts contain moderate mixed inflammatory infiltrates with interface activity (H&E, 10×). b) The portal tracts show lymphocytes with admixed clusters of plasma cells (H&E, 20×). c) The hepatic lobule shows aggregates of inflammatory cells with apoptotic bodies (H&E, 20×). d) Hepatic lobule with mild hepatocanalicular cholestasis (H&E, 40×). Black arrows point to key histological features described in the subfigures.

Her Roussel Uclaf Causality Assessment Method (RUCAM) score was calculated to be six, suggesting DILI as a probable cause of her presentation [15]. A liver biopsy was subsequently performed. Portal inflammatory infiltrates with predominant lymphocytes and mixtures of plasma cells and eosinophils, moderate interface activity, bile ductular proliferation, and lobular disarray with scattered apoptotic bodies were observed (Figure 1). Collectively, these findings were suggestive of DILI with autoimmune features [16,17]. The patient endorsed use of an herbal supplement with ingredients not associated with DILI, including lavender, cedarwood, and coriander. Her medications, metformin, insulin, and simvastatin, are not commonly associated with DILI. However, she reported an 11-day course of AC, which she had completed about 70 days before her presentation. Given the strong association of AC with DILI, this was presumed to be the most likely cause of her condition.

She was started on high-dose intravenous steroids (methylprednisolone), and her liver function tests (LFTs) and symptoms began to improve significantly. She was later deemed stable for discharge with oral steroids (prednisone) and close outpatient follow-up. In the outpatient setting, her prednisone was gradually tapered. However, her LFTs showed two relapses at six weeks and 11 months following discharge, respectively, prompting increases in prednisone dosage and the addition of long-term mycophenolate treatment for suspected AIH (Table 3). Two well-accepted clinical diagnostic tools by Hennes et al. [18] (a modified version of the International Autoimmune Hepatitis Group (IAIHG) score) and Alvarez et al. [19] (IAIHG score) showed the patient to have scores of seven and 22, respectively, suggesting definite AIH.

**Table 3:** Timeline showing changes in liver function tests with two relapses and subsequent adjustments in immunosuppressive treatment during 13 months of monitoring in our patient Values in bold are elevated. *Prednisone dose was tapered from 40 to 30, 30 to 7.5, and 15 to 5 mg by stepwise decreases of 2.5-5 mg approximately every two weeks (not shown) after three months.

Lab Test/Medication	Discharge	Four Weeks	Six Weeks	Seven Weeks	Three Months	Six Months	11 Months	13 Months	Reference Range
Alanine Aminotransferase (IU/L)	238	31	130	165	15	6	127	7	0-44
Aspartate Aminotransferase (IU/L)	128	34	87	110	14	10	105	11	0-40
Prednisone Dose (mg)	40	30	40	40	30*	7.5*	15	5*	N/A
Mycophenolate Dose (mg)	-	-	-	1000	1000	1500	1500	1500	N/A

## Discussion

This case highlights a unique presentation of AC-induced DILI unraveling AIH in a previously asymptomatic female. AIH has rarely been reported following DILI and often resolves without relapse [6,7]. Our patient experienced relapses requiring long-term immunosuppression and demonstrated unique clinical and pathological findings of AIH following AC-induced DILI, an occurrence which has only rarely been reported following AC exposure in the literature [12,13].

It is difficult to say whether this patient had DIAIH or CAIH, as these conditions share many features. However, certain key differences in clinical and laboratory presentations between DIAIH and cAIH may be noted. DIAIH may present within days of drug exposure, while cAIH more often presents months after the use of an implicated drug [6]. Additionally, while DIAIH may present with a mixed hepatocellular or cholestatic pattern of liver injury, cAIH has a stronger predisposition for elevated hepatocellular markers only [6,20]. High ANA titers are more commonly observed in cAIH than DIAIH [7,11]. In DIAIH, there is often a response to cessation of the causative agent alone and a relatively low risk of recurrence, while cAIH typically requires long-term immunosuppression with steroids, mycophenolate, and/or azathioprine to prevent relapse [7,21,22]. In our case, the patient exhibits some features of cAIH: the latency of several months of her presentation following exposure to AC, the hepatocellular pattern of her liver injury, the significantly elevated ANA titer, and the occurrence of relapses necessitating the need for long-term immunosuppressant therapy.

Additionally, certain differences in histopathology between DIAIH and cAIH may be observed. In a study led by Alkashah et al. [10], liver biopsies of a small cohort of 15 patients showed greater plasma cell infiltration and interface hepatitis in cAIH than DIAIH patients, a distinguishing feature for diagnosis. Additionally, Suzuki et al. [20] reported features such as severe portal inflammation, prominent plasma cell infiltrate, and focal necrosis as features supportive of cAIH. Notably, all these features were observed in our patient’s liver biopsy, supporting a possible diagnosis of cAIH. Combining these histological features with clinical features such as a history of autoimmune disease (Hashimoto’s thyroiditis), a highly elevated ANA titer, and relapses requiring extended immunosuppressive therapy, our patient was shown to have definite AIH [18,19].

## Conclusions

In conclusion, our report highlights a case of AC-induced DILI with AIH as supported by clinical and biopsy findings. AC is a very common cause of DILI, yet AIH is not a common association. Since they share many features, it is challenging to conclude whether our patient developed DIAIH or cAIH after exposure to AC. However, since most cases of DIAIH resolve after withdrawal of the offending agent, our patient’s case is unique in that she experienced relapse and required long-term immunosuppressive treatment for more than a year after AC exposure. In this case, perhaps AC was simply the straw that broke the camel’s back, unraveling AIH, requiring long-term immunosuppression in a previously asymptomatic female.
